# DeepTraSynergy: drug combinations using multimodal deep learning with transformers

**DOI:** 10.1093/bioinformatics/btad438

**Published:** 2023-07-19

**Authors:** Fatemeh Rafiei, Hojjat Zeraati, Karim Abbasi, Jahan B Ghasemi, Mahboubeh Parsaeian, Ali Masoudi-Nejad

**Affiliations:** Department of Epidemiology and Biostatistics, School of Public Health, Tehran University of Medical Sciences, Tehran 1417613151, Iran; Department of Epidemiology and Biostatistics, School of Public Health, Tehran University of Medical Sciences, Tehran 1417613151, Iran; Laboratory of System Biology, Bioinformatics & Artificial Intelligent in Medicine (LBB&AI), Faculty of Mathematics and Computer Science, Kharazmi University, Tehran 1571914911, Iran; Chemistry Department, Faculty of Chemistry, School of Sciences, University of Tehran, Tehran 1417614411, Iran; Department of Epidemiology and Biostatistics, School of Public Health, Tehran University of Medical Sciences, Tehran 1417613151, Iran; Department of Epidemiology & Biostatistics, School of Public Health, Imperial College London, London W21PG, United Kingdom; Laboratory of Systems Biology and Bioinformatics (LBB), Institute of Biochemistry and Biophysics, University of Tehran, Tehran 1417614411, Iran

## Abstract

**Motivation:**

Screening bioactive compounds in cancer cell lines receive more attention. Multidisciplinary drugs or drug combinations have a more effective role in treatments and selectively inhibit the growth of cancer cells.

**Results:**

Hence, we propose a new deep learning-based approach for drug combination synergy prediction called DeepTraSynergy. Our proposed approach utilizes multimodal input including drug–target interaction, protein–protein interaction, and cell–target interaction to predict drug combination synergy. To learn the feature representation of drugs, we have utilized transformers. It is worth noting that our approach is a multitask approach that predicts three outputs including the drug–target interaction, its toxic effect, and drug combination synergy. In our approach, drug combination synergy is the main task and the two other ones are the auxiliary tasks that help the approach to learn a better model. In the proposed approach three loss functions are defined: synergy loss, toxic loss, and drug–protein interaction loss. The last two loss functions are designed as auxiliary losses to help learn a better solution. DeepTraSynergy outperforms the classic and state-of-the-art models in predicting synergistic drug combinations on the two latest drug combination datasets. The DeepTraSynergy algorithm achieves accuracy values of 0.7715 and 0.8052 (an improvement over other approaches) on the DrugCombDB and Oncology-Screen datasets, respectively. Also, we evaluate the contribution of each component of DeepTraSynergy to show its effectiveness in the proposed method. The introduction of the relation between proteins (PPI networks) and drug–protein interaction significantly improves the prediction of synergistic drug combinations.

**Availability and implementation:**

The source code and data are available at https://github.com/fatemeh-rafiei/DeepTraSynergy.

## 1 Introduction

It is shown that multidisciplinary drugs or drug combinations have more effective treatments than single-drug therapies and selectively inhibit the growth of cancer cells (Tang 2017). Designing new drugs with optimal performance for cancer patients is highly important in the pharmaceutical industry (He *et al.* 2018, Lee *et al.* 2018, Abbasi *et al.* 2019, Abbasi 2021). The primary purpose of such studies is to prioritize combination anticancer therapies based on the simultaneous use of several drugs with different mechanisms of action to overcome the resistance of single medicines and reduce side effects (Pemovska *et al.* 2013, Masoudi-Sobhanzadeh *et al.* 2021). It is revealed that several target genes are involved in cancer cell proliferation, like genomic complexity and molecular contexts, and heterogeneity of tumors. Furthermore, their protein products are essential in controlling abnormal pathways and networks that lead to diverse responses to anticancer drugs among patients (Pang *et al.* 2014, Rubin 2015, Schmitt *et al.* 2016).

Drug combinations have emerged as a promising therapeutic approach to overcoming drug resistance and improving the effectiveness of anticancer therapies (Wang *et al.* 2021). In other words, it can use multiple drugs to aim at various targets, pathways, or cellular processes involved in the pathogenesis of a particular disease (Anighoro *et al.* 2014, Madani Tonekaboni *et al.* 2018, Masoudi-Sobhanzadeh 2020). The number of possible drug combinations increases rapidly with increasing the number of drugs; hence, wet-experimental methods are not enough to discover new drug compounds. Therefore, to reduce the search space for drug combinations, there is a need to develop more efficient computational methods for predicting synergistic drug compounds (Paul *et al.* 2010, Wu *et al.* 2021).

Current computational methods use synergistic scores to predict effective drug combinations. The synergistic score is defined as the degree of drug interactions. Synergy is generally determined by a selected reference model based on the properties of dose–response curves, which measure the response rate based on the difference between expected and observed dose–response profiles (Zagidullin *et al.* 2021). Subsequently, this combination can be classified as synergistic, additive, or contrasting. Information such as structural similarity and biochemical properties is vital to understanding drug compounds’ behavior. Also, incorporating drug–target and drug–drug interactions can improve effective combination therapies (Masoudi-Nejad *et al.* 2013, Mousavian *et al.* 2016). Moreover, drug–protein interaction and protein–protein interaction (PPI) are important factors for investigating the effectiveness of drug synergy (Chen *et al.* 2016, Chen *et al.* 2020, Wang *et al.* 2021). In recent years, the expression profile of genes has also helped to predict the synergistic effects of drug compounds on cancer cell lines (Preuer *et al.* 2018, Guo *et al.* 2021, Dehghan *et al.* 2023).

Up to now, many approaches to drug synergy prediction are introduced. Some of these approaches use classic machine learning methods. Sidorov *et al.* (2019) used Random Forest (RF) and Extreme Gradient Boosting (XGBoost) machine learning techniques to predict the best synergy of a given drug combination by cell line. Their results show that XGBoost offers slightly better performance than the RF method. Julkunen *et al.* (2020) provided a new machine learning framework for predicting drug combination responses in preclinical studies based on cell lines or patient-derived cells called comboFM. They applied a higher-order factorization machine (FM) to learn the higher-order tensors of drug pairs. The input features of comboFM contained two molecular fingerprints of the drugs, concentration values of both drugs and gene expression profiles of cancer cell lines. It is shown that ComboFM is an effective tool for systematically prescreening drug compounds to support accurate oncology programs. Chen *et al.* (2016) introduced a novel semisupervised algorithm that not only uses the drug’s chemical structure but also utilizes a drug–target interaction network. Liu *et al.* (2019) inspired the drug–protein heterogeneous network-based inference to derive the properties of the drug combinations. They trained the gradient tree boosting classifier to predict new drug combinations using the extracted properties. Zhang and Yan (2019) developed a field-aware FM that incorporates pharmacological data into predicting two- and three-drug synergistic combinations.

In recent years, deep learning-based drug synergy prediction has received attention. Some of these approaches only utilize the knowledge of proteins directly targeted by drugs and diseases. Yang *et al.* (2021) introduced an advanced chart-based deep learning method that used the graphical representation of the PPI network to identify anticancer drug combinations. Using a space-based convolution network, the model encodes information about the topological structure of the target protein modules of a drug pair and the protein modules associated with a particular cancer cell line in the PPI network. Kim *et al.* (2021) used a drug synergistic prediction model based on multitask deep artificial neural networks in understudied cancer types. To overcome the data scarcity challenge, they have utilized transfer learning techniques. As a result, models trained on data-rich tissues are transferred to data-poor tissues. They also utilize a multitask deep neural network with multimodal inputs (molecular, genetic, phenotypic characteristics) and multiple outputs (drug sensitivity and synergism) for cancer cell lines. Zhang *et al.* (2021) used the autoencoder’s deep neural network (AuDNN) model to predict the synergy of drug combinations using the integration of multiomics data and the chemical structure of the data. Three autoencoders were trained to generate representations of cancer cell lines from gene expression, copy number, and genetic mutation data of tumor samples. AuDNNsynergy can also be used to predict combinations in novel cell lines.

Jiang *et al.* (2020) used a graph convolution network to predict the synergy of the drug combination in 39 cell lines derived from six types of cancer. Multimodal graphs were constructed for each cell line based on the drug–drug interaction networks, drug–protein interaction networks, and PPI networks. Jin *et al.* (2021) developed a deep learning-based model using ComboNet that jointly uses molecular structure and biological targets to predict synergistic drug combinations. Brahim Kuru *et al.* (2021) presented a deep neural network-based algorithm for predicting synergistic drug scores using drug chemical structure information and cell line gene expression called MatchMaker. Dong *et al.* (2021) introduced an interpretable deep signaling pathway called IDSP, which is a deep diagram neural network. In their work, gene–gene and gene–drug regulatory relationships are included in synergistic drug prediction. Li *et al.* (2023) utilize the Siamese convolutional network and random matrix projection to learn more informative drug combination features. Then, after extracting cell line features by using the convolutional network, these features are integrated and passed into multilayer perceptron (MLP) to predict the synergy score.

Liu and Xie (2021) introduced a knowledge-based deep learning model, TranSynergy, and a new method for enrichment analysis of the Shapley additive gene complex (SA-GSEA) to predict synergistic drug combinations and improve the interpretability of the machine learning model. Wang *et al.* (2021) proposed a deep learning-based model that uses graph neural networks and an attention mechanism to predict the synergy of drug combinations. Sun *et al.* (2020) proposed a deep tensor factorization model that combines a framework based on tensor factoring and a deep neural network to predict the synergistic effect of drug pairs. It achieves almost as good predictive performance as the advanced model (DeepSynergy) while using significantly fewer data sources.

In multitask learning (MTL), several tasks are learned and predicted simultaneously. MTL improves the prediction accuracy of each task-specific model compared to training each model separately. This study proposes a new deep multitask and multimodal approach for drug synergy prediction. The model gets PPI, cell–target interaction, and both drug sequences as input and predicts the drug–target interaction, its toxic effect, and drug combination synergy as different tasks. In our approach, drug combination synergy is the main task and the two other ones are the auxiliary tasks that help the approach to learn a better model. Our approach is different from the other state-of-the-art protocols as follows:

1. To describe drug molecules, we propose a transformer-based approach.
2. To predict the interaction between input drugs and all protein sequences, we use a binding affinity prediction model. A one-class learning loss is used to learn only active compound–target pairs.
3. We propose a new architecture that effectively combines drug–target interaction, PPI, and cell–target interaction to incorporate drug synergy prediction.
4. We propose a toxic loss to prevent overlapping exposure. Overlapping exposure happens when the drug–target modules overlap with each other and the disease modules.
5. We also conducted comprehensive ablation studies to validate the significance of the different modules of the proposed approach.

The paper is organized in the following manner. Section 2 describes in detail the proposed method. The experimental results are presented in Section 3. Finally, the discussion and future work are provided in Section 4.

## 2 Materials and methods

**Figure 1.**
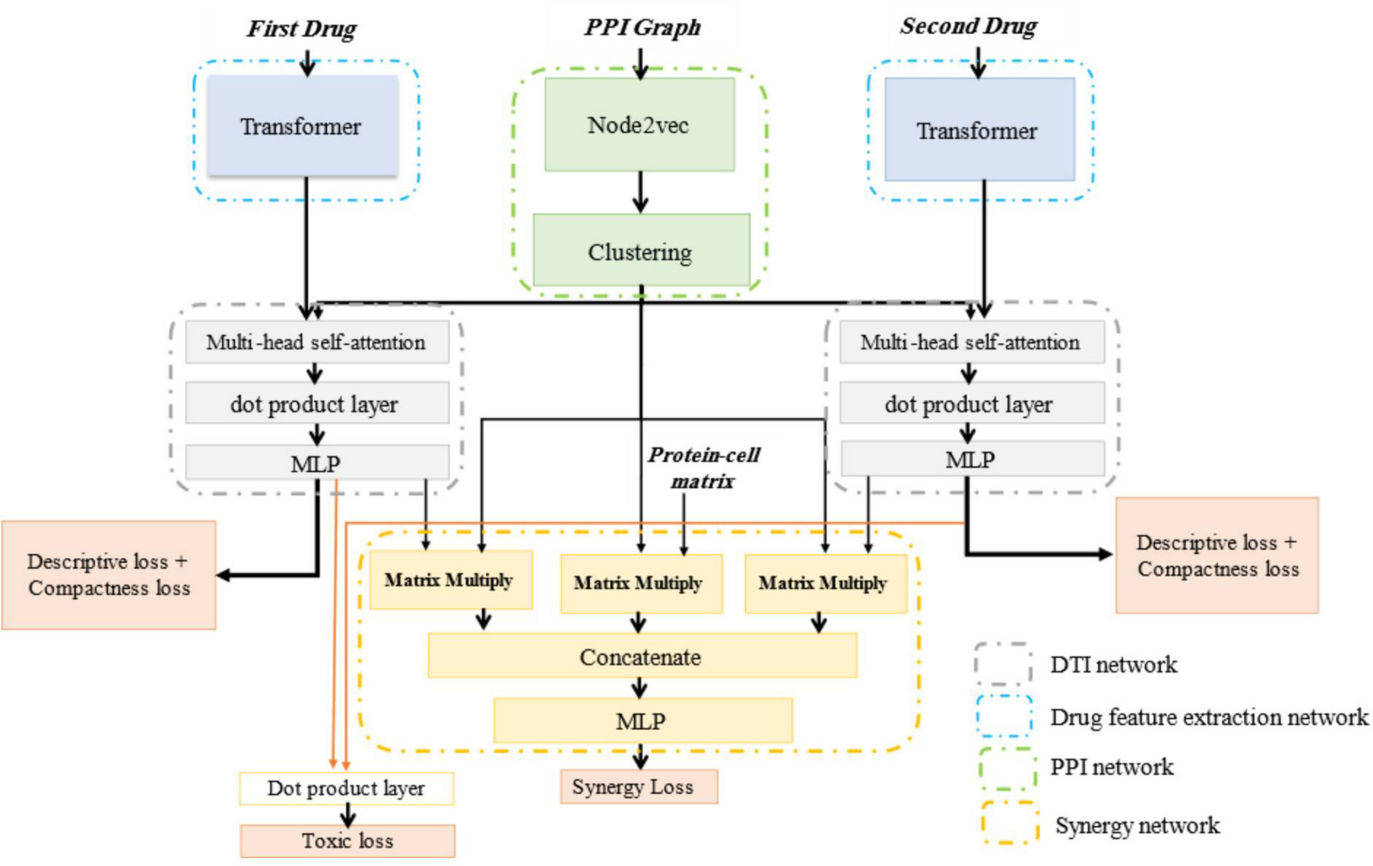
The overall schematic of the proposed approach. All of the inputs of the network are shown as bold and italic text.

The overall schematic of the proposed approach is depicted in Fig. 1. As it is mentioned in Section 1, the aim is to predict the synergy of the pair drug combination based on protein–protein, drug–protein, and cell line–protein interactions. Generally, the proposed deep learning-based architecture has four main subnetworks: a PPI network, a drug feature extraction network, a protein–compound interaction network, and a synergy prediction network.

At first, the problem formulation is presented, followed by a detailed explanation of each step of the proposed approach.

### 2.1 Problem formulation

Given ${\left\{\left(d_{1}^{\left(i\right)},d_{2}^{\left(i\right)},c^{\left(i\right)}\right),s^{\left(i\right)}\right\}}_{i=1}^{N}$ where $d_{1}^{\left(i\right)}$ and $d_{2}^{\left(i\right)}$ denote the first and second drugs of the ith drug pair sample. The synergy value of the paired drug for a cell line (denoted by $c^{\left(i\right)}$) is represented by $s^{\left(i\right)}$. The main goal is to design a system to predict the synergistic value of the input-paired drugs. To this end, the following items are defined as the input to the approach:

- A pair of drugs: $\left(d_{1}^{\left(i\right)},d_{2}^{\left(i\right)}\right)$
- A cell line: $c^{\left(i\right)}$
- PPI graph: $G_{\mathrm{pp}}$
- Protein–cell matrix: $C_{\mathrm{pc}}$

where $G_{\mathrm{pp}}$ and $C_{\mathrm{pc}}$ are used as auxiliary inputs of the approach. In the $G_{\mathrm{pp}}$, each node is a protein, and the connection between two proteins exists if there are biochemical events and/or electrostatic forces between them. Set $C_{\mathrm{pc}}$ is a matrix with $\left|P\right|\times \left|C\right|$ elements where $\left|P\right|$ and $\left|C\right|$, respectively, denote the number of proteins and the number of cells.

Synergy score $(s^{\left(i\right)}$) is computed based on the zero-interaction potency (ZIP) reference model. The ZIP model captures the drug interaction relationships by comparing the changes in the potency of the dose–response curves between individual drugs and their combinations. By combining the advantages of both the Loewe and the Bliss models, the ZIP model assumes that two noninteracting drugs are expected to incur minimal changes in their dose–response curves (Liu *et al.* 2020).

### 2.2 PPI network

In the proposed approach, the node2vec network is utilized to analyse the PPI network. To this end, node2vec is utilized to learn a representation for each node of $G_{\mathrm{pp}}$. The node2vec network which is shown by $N_{P}$ produces a representation for each protein based on its neighborhood in the $G_{\mathrm{pp}}.$ It should be noted that the neighborhood of each node is preserved in the learned representation. This learned representation can lead to improving the predictive power.

To reduce the number of proteins, the output of the nod2vec, the learned feature vector of proteins, is fed into a clustering method, a k-means algorithm, to generate a group of proteins with the same feature representation. The reason for this is that the number of unique proteins is high. Hence it leads to computational issues in the proposed approach. In node2vec, it is expected proteins with the same local neighborhood have the same feature vector. Therefore, by clustering, these proteins are placed in the same cluster. The number of clusters is determined experimentally. The final output of clustering is $o_{p}\in R^{\left|P_{c}\right|\times n}$ where $\left|P_{c}\right|$ is the number of protein clusters and *n* is the dimension of the representation space.

### 2.3 Drug feature extraction network

**Figure 2.**
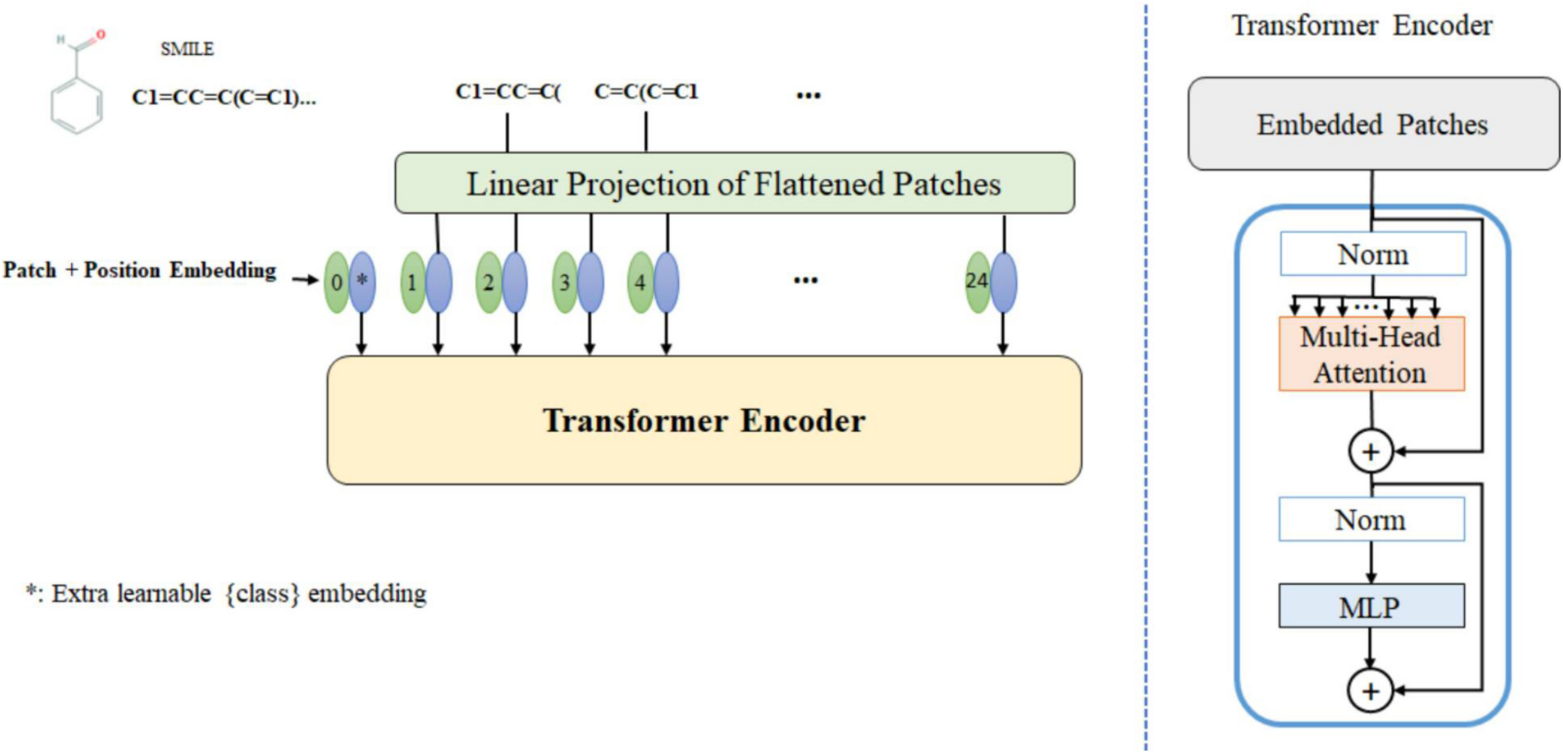
The overall schematic of the Transformer is used as a feature extractor in the proposed approach.

The proposed approach introduces an architecture based on transformers to extract features from drugs (Fig. 2). One of the main advantages of a transformer that lead us to utilize it is that it provides context for any position in the drug molecule. Also, the parts of the drug molecule which have most efficient in predicting, get the higher importance. The vision transformer, introduced by Dosovitskiy *et al.* (2020), is modified to apply to the sequence data derived from the SMILES representation of the drug molecule. To this end, at first, the SMILES representation of the drug molecule is divided into some patches. To prevent missing information, the patches should overlap by choosing an appropriate segment length. Then, each patch is fed into the embedding layer, including patch embedding and position embedding. The transformer encoder consists of a normalization layer, a multihead attention layer, and a MLP. The transformer encoder is repeated L times in the architecture to create the feature extraction network. A feature extraction network is shared between two drugs in the proposed approach. Hence for the feature representation of the drugs, the output of $N_{F}$ network, for the first and second drugs of the input, are, respectively, shown by $o_{F}^{d_{1}}$ and $o_{F}^{d_{2}}$.

### 2.4 Compound–protein interaction network

In this section, the compound–protein interaction network is explained. It should be noted that many compound–protein interactions are not yet discovered. Hence, in designing this network, this issue is considered. The labeled compound–protein pairs are limited, and the available pairs are the active ones. All known inactive pairs and unknown pairs are considered inactive. Therefore, the loss function for the protein–compound interaction network should be learned with samples that all of them are active. To consider this issue, we utilize one-class classification loss. In machine learning, one-class classification gets more attention (Perera and Patel 2019). This paper uses compactness loss and descriptiveness loss, introduced by Perera and Patel (2019).

The inputs of this subnetwork are the feature representation of the drug $o_{F}^{d_{li}},\;l=1,2,$ and the feature representation of all proteins $o_{p}$. Compound–protein interaction network includes a multihead self-attention layer, a dot product layer, and an MLP. Multihead self-attention layer computes an attention mechanism several times in parallel. It is effective because it could jointly consider multiple positions of importance. MLP gets the $o_{p}$ as input and maps it to a new representation ($o_{p}^{m}$) with the same distribution as the drug representation space. Moreover, if a drug can bind to some proteins to form a drug–protein complex, protein and compound binding sites are expected to have the same representation. Hence, $o_{F}^{d_{li}}$ and $o_{p}^{m}$ are fed into the dot product layer to produce a binding affinity of drug l with $P_{c}$ proteins. The output of this subnetwork is a matrix represented by $o_{B}^{d_{li}}$. To effectively learn the compound-protein interaction network, the descriptiveness loss ($L_{\mathrm{Descriptive}}$) is defined using the cross-entropy loss which states the model’s ability to discriminate the different classes. In our work, similar to Perera and Patel (2019), the compactness loss ($L_{\mathrm{compactness}}$) is measured by the variance of each feature batch. It means that the feature vector of all samples which belong to the same class should be similar.

#### 2.4.1 Synergy network 

The inputs of the synergy network are $o_{F}^{d_{1i}}$ and $o_{F}^{d_{2i}}$, $o_{B}^{d_{1i}}$ and $o_{B}^{d_{2i}}$, $C_{\mathrm{pc}}$, and $c^{\left(i\right)}$. As it is shown, it contains several dot product layers. As the first step, $o_{B}^{d_{li}}$ and $o_{p}^{m}$ are fed into the dot product layer. This layer aims to calculate the representation of all proteins that the first drug can bind. A similar computation is done for the second drug. The outputs of the product layers for this pair of drugs are concatenated and fed into an MLP. The output of the MLP network is shown by $o_{\mathrm{BM}}$. In the second step, $o_{B}^{d_{li}}$ and the corresponding vector of $c^{\left(i\right)}$ in $C_{\mathrm{pc}}(c^{\left(i\right)})$ are fed into the dot product layer. Its goal is to consider the representation of the related signaling proteins of the input cell line. The output of the two-dot product layers is concatenated and fed into an MLP network whose output is shown by $o_{\mathrm{CM}}$. Finally, $o_{\mathrm{BM}}$ and $o_{\mathrm{CM}}$ are concatenated and fed into the last MLP layers to predict the synergy value. In the proposed approach, fusion is done in the class. The final synergy value for the *i*th drug pairs is shown by o. The synergy loss function is a binary cross-entropy loss which is defined as follows:


(1) $$L_{\mathrm{synergy}}=\sum_{i}\left[\left(s^{\left(i\right)}\log\left(o_{\;}^{\left(i\right)}\right)\right)+\left(\left(1-s^{\left(i\right)}\right)\log\left(1-o_{\;}^{\left(i\right)}\right)\right)\right].$$

In drug synergy prediction, an effective approach should consider the toxic effect originating from overlapping exposure. It has been shown that overlapping exposure is statistically significant in the occurrence of adverse effects. Hence, we define another term in the overall loss function which considers this issue. Each drug is expected to have separated bindable target proteins to prevent toxicities (Cheng *et al.* 2019, Yang *et al.* 2021). Therefore, the toxic loss function ($L_{\mathrm{Toxic}}$) is defined as follows: $L_{\mathrm{Toxic}}$ is minimized when the inner product of $o_{B}^{d_{1i}}$ and $o_{B}^{d_{2i}}$ to be zero. It happens if the first and second drugs have separated bindable target proteins.


(2) $$L_{\mathrm{Toxic}}={\left(o_{B}^{d_{1}}\right)}^{T}o_{B}^{d_{2}}.$$

In our work, the whole loss is defined as follows: where $\lambda_{1}$, $\lambda_{2}$, and $\lambda_{3}$, respectively, are the weights of the toxic loss, compactness loss, and descriptive loss in the final loss.


(3) $$L=L_{\mathrm{synergy}}+\lambda_{1}L_{\mathrm{Toxic}}+{\lambda_{2}L}_{\mathrm{compactness}}+\lambda_{3}L_{\mathrm{descriptive}},$$

## 3 Experiments

### 3.1 Dataset

The approach is applied to two well-known datasets, including DrugCombDB (Liu *et al.* 2020) and OncologyScreen (O’Neil *et al.* 2016). DrugCombDB is a comprehensive dataset of drug combinations that are collected from many different resources like high throughput screening, an external database, or manual curation from PubMed literature. DrugCombDB contains 6 891 566 drug pairwise combinations with 2887 unique drugs and 124 unique cell lines. In our approach, similar to other competing approaches, we used a shortened version of the DrugCombDB dataset. It contains 69 436 drug pairwise combinations with 764 unique drugs and 76 unique cell lines. Oncology-Screen is a smaller dataset in drug combinations. It contains 4176 drug pairwise combinations with 21 unique drugs and 29 unique cell lines.

In both datasets, we perform 5-fold cross-validation. To do so, we divide the whole dataset into five equal parts, and then in each run, we consider the four parts as training and validation data, and the remaining part is considered as test data. This procedure is repeated five times and finally, the average performance is reported.

### 3.2 Performance measures

To describe the performance of the predictive model, performance is evaluated by measures that are common for classification tasks: the area under the receiver operating characteristic curve (AUC-ROC), the area under the precision–recall curve (AUC-PR), accuracy (ACC), recall, and F1 score. These measures are selected to address different characteristics of the learned models.

In the whole experiment, hyperparameter optimization is done using grid search. This optimization is done for the number of clusters search over {200, 300, 400, 600}, learning rate search over {0.0001, 0.001, 0.01, 0.1}, the weights of the toxic loss, compactness loss, and descriptive loss ($\lambda_{1}$, $\lambda_{2}$, and $\lambda_{3}$) search over {$\frac{1}{4}$, $\frac{1}{2}$, 1}, the patch size search over {20, 30, 40, 50, 60}, and the overlap stride ratio search over {$\frac{1}{4}$, $\frac{1}{3}$, $\frac{1}{2}$, $\frac{3}{4}$}. The overlap stride ratio r is defined to be the ratio of the patch size that overlapped with the neighboring patch.

### 3.3 Ablation study

To show the impact of the different modules on the proposed approach’s performance, an ablation study is done. To do so, three versions of the proposed approach are created: (i) Transformer: in this version, only the synergy loss is considered. (ii) Transformer+Toxic: in this version, synergy and toxic losses are considered, and (iii) Transformer+DTI: in this version, synergy and interaction losses are considered.

The obtained results of the ablation study applied to DrugCombDB and OncologyScreen datasets are shown in Tables 1 and 2, respectively. As it is shown in Table 1, the proposed approach (DeepTraSynergy), which comprises synergy, toxic and interaction losses, gets the best results according to the considered performance metrics. From the ablation study, it can be concluded that the contribution of each component (i.e. synergy loss, toxic loss, interaction loss) brings further improvement.

**Table 1.** The obtained result of the ablation study on the DrugCombDB dataset.

Model	ACC	Recall	AUC-ROC	AUC-PR	F1	*P*-value *t*-test
Transformer	0.6802	0.7597	0.7859	0.7926	0.7205	4.35 × 10^−3^
Transformer+Toxic	0.7023	0.6944	0.7189	0.7308	0.6705	3.63 × 10^−4^
Transormer+DTI	0.7043	0.6785	0.7605	0.7725	0.6911	1.43 × 10^−3^
**DeepTraSynergy**	**0.7715**	**0.8012**	**0.8321**	**0.8487**	**0.7608**	

The bolded items in the table are the performance measure of our model.

**Table 2.** The obtained result of the ablation study on the OncologyScreen dataset.

Model	ACC	Recall	AUC-ROC	AUC-PR	F1	*P*-value *t*-test
Transformer	0.7611	0.8023	0.8111	0.8205	0.7398	3.81 × 10^−4^
Transformer+Toxic	0.7588	0.8095	0.8144	0.8204	0.7412	6.14 × 10^−4^
Transformer+DTI	0.7773	0.8198	0.8378	0.8515	0.7741	9.26 × 10^−5^
**DeepTraSynergy**	**0.8052**	**0.8491**	**0.8637**	**0.8818**	**0.8112**	

The bolded items in the table are the performance measure of our model.

From Table 2, DeepTraSynergy achieves the best performance in all five measures including ACC, Recall, AUC-ROC, AUC-PR, and F1-score. Table 2 shows that by adding drug–target interaction knowledge (Transformer+DTI), ACC gets a 1.62% improvement over the Transformer. DeepTraSynergy improves ACC even more (by 4.41%) that confirms each module of the proposed approach contributes to the performance improvement. To statistically evaluate the significant improvement of the different modules of the proposed approach, the *t*-test is utilized at a significant level of 0.05. The obtained value of *P*-value in Tables 1 and 2 shows that DeepTraSynergy (full proposed approach) outperforms the other approaches.

### 3.4 Method comparison

To show the effectiveness of the proposed approach, three state-of-the-art approaches including Grarep (Cao *et al.* 2015), DeepSynergy (Preuer *et al.* 2018), GraphSynergy (Yang *et al.* 2021), NEXGB (Meng *et al.* 2022), and KGNN (Lin *et al.* 2020) are selected for comparison in DrugCombDB and OncologyScreen datasets (Tables 3 and 4, respectively). As it is shown, in Tables 3 and 4, our approach gets the best results with respect to KGNN, GCN, and DeepSynergy methods. Compared with the GraphSynergy method, the values AUC-ROC for the DrugCombDB dataset is reduced. In DrugCombDB, we get a 3.27% improvement in the AUC-PR measure over the best comparing method. It means our approach cares more about the positive class than the other approaches. The presented DeepTraSynergy gets the most improvement in recall measure. From these results, it can be inferred that the proposed approach effectively lowers the number of false-negative samples. It causes the proposed DeepTraSynergy method outperforms GraphSynergy and NexGB for the prediction of the synergic drug pairs. We have done *t*-test to statistically evaluate the proposed approach respect to the other approaches. The obtained results show that on DrugCombDB dataset, DeepTraSynergy outperforms five approaches at a significant level of 0.05 and compared with GraphSynergy and NEXGB outperforms with a *P*-value of lower than .1. Also, on oncologyScreen dataset, DeepTraSynergy outperforms all other approaches with a *P*-value lower than .02.

**Table 3.** The comparison of DeepTraSynergy with the comparable approaches on the DrugCombDB dataset.

Model	ACC	Recall	AUC-ROC	AUC-PR	F1	*P*-value *t*-test
KGNN	0.6602	0.6751	0.7241	0.6916	0.6441	1.52 × 10^−4^
GCN	0.6733	0.6015	0.7211	0.6905	0.6264	1.48 × 10^−3^
GraRep	0.667	0.581	0.728	0.689	0.613	2.34 × 10^−3^
DeepSynergy	0.6846	0.6256	0.7461	0.7296	0.6434	1.99 × 10^−3^
DeepDDS^a^	0.7312	0.7219	0.7985	0.7814	0.6914	2.84 × 10^−3^
GraphSynerg	0.7545	0.7184	**0.8351**	0.8160	0.7281	8.43 × 10^−2^
DFFNDDS	0.681	0.360	0.651	0.489	0.416	1.15 × 10^−2^
NEXGB	0.762	0.704	0.833	0.811	0.729	1.09 × 10^−1^
**DeepTraSynergy**	**0.7715**	**0.8012**	0.8321	**0.8487**	**0.7608**	

a We run the approach by ourselves.

The bolded items in the table are the performance measure of our model.

**Table 4.** The comparison of DeepTraSynergy with the comparable approaches on the OncologyScreen dataset.

Model	ACC	Recall	AUC-ROC	AUC-PR	F1	*P*-value *t*-test
KGNN	0.7080	0.7562	0.7820	0.7718	0.7280	5.52 × 10^−5^
GCN	0.6615	0.7375	0.7146	0.7096	0.6926	2.21 × 10^−4^
GraRep	0.663	0.767	0.723	0.702	0.702	1.38 × 10^−3^
DeepSynergy	0.6898	0.7179	0.7664	0.7696	0.7052	3.68 × 10^−5^
DeepDDS^a^	0.7108	0.6302	0.7158	0.7498	0.7012	2.87 × 10^−3^
GraphSynerg	0.7635	0.8008	0.8450	0.8497	0.7792	2.33 × 10^−3^
DFFNDDS	NA	NA	NA	NA	NA	—
NEXGB	0.782	0.827	0.858	0.873	0.804	2.45 × 10^−2^
**DeepTraSynergy**	**0.8052**	**0.8491**	**0.8637**	**0.8818**	**0.8112**	

a We run the approach by ourselves.

The bolded items in the table are the performance measure of our model.

From the results of Tables 2 and 4, it is found that a simple architecture in which a transformer is used as a feature extractor (i.e. Transformer) could get comparable results with respect to the other methods denoting that the Transformer can learn more discriminative features.

**Figure 3.**
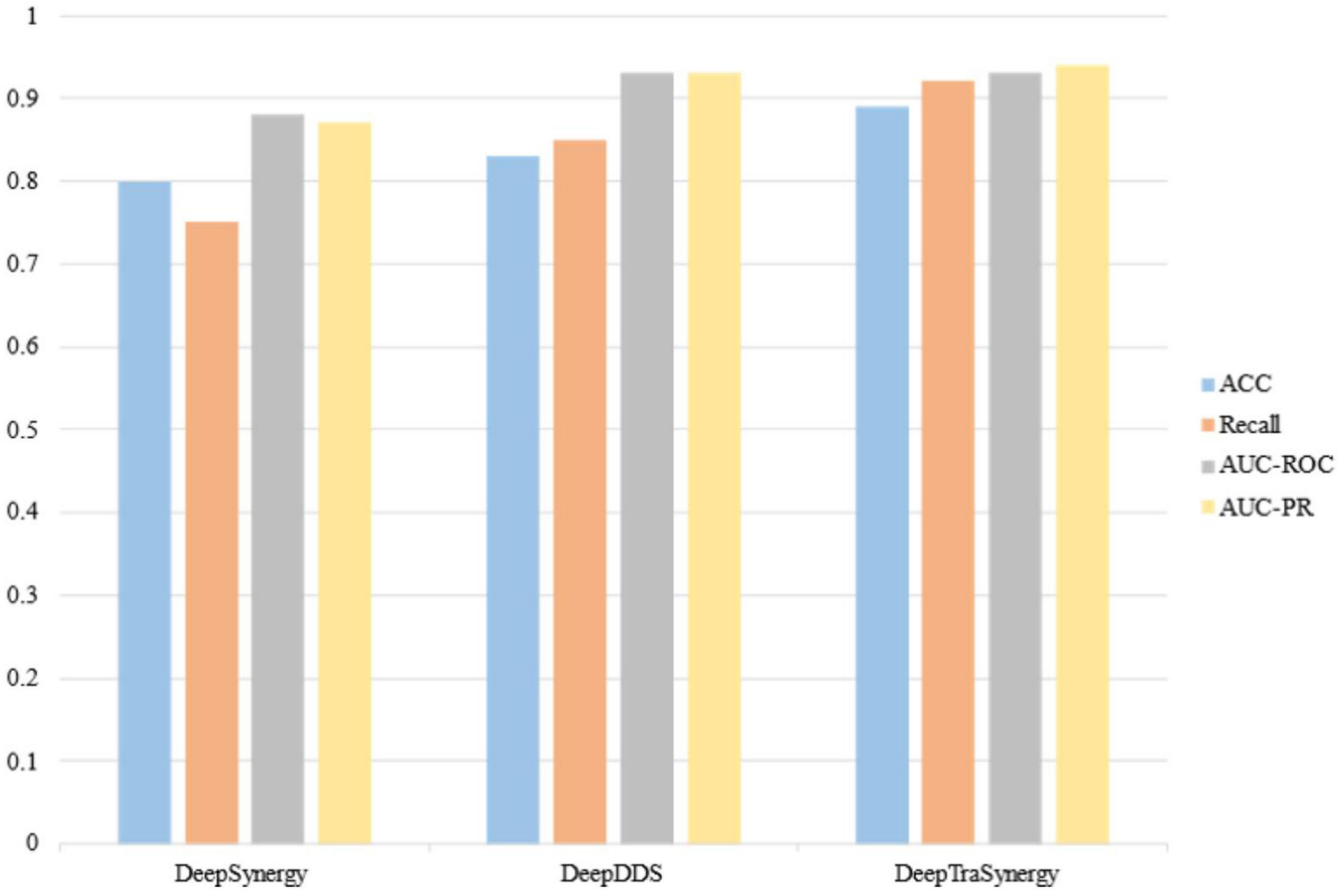
The comparison of DeepTraSynergy with the comparable approaches on the O’Neil dataset.

It should be noted that the OncologyScreen dataset is a subset of the O’Neil dataset. In other words, the OncologyScreen dataset contains 4176 pairwise drugs and cell line combinations with 21 unique drugs and 29 unique cell lines. While, the O’Neil dataset, not only contains all two drug-cell triplets of the OncologyScreen dataset but also contains other additional samples. To recap, there are 13 243 unique pairwise drugs and cell line combinations, consisting of 38 drugs and 31 cell lines. It should be noted that the original dataset contains 23 052 drug pairs and cell lines. After removing replicated ones like Wang *et al.* (2021) and Hu *et al.* (2022), we have 13 243 triplets. Also, like Wang *et al.* (2021) and Hu *et al.* (2022), we have used 10 as a threshold to classify the triplets as synergistic and nonsynergistic ones. The obtained results are shown in Fig. 3. As it is shown, DeepTraSynergy gets better results in all measures.

#### 3.4.1 Investigate the effect of patch size

To investigate the effect of patch size and overlap stride ratio in the final performance of the proposed approach, we have experimented. To do so, we have done an experiment with the best-chosen hyperparameter values for the learning rate (0.001) and the number of clusters (300). Then, we change the patch size search over {20, 30, 40, 50, 60} and the overlap stride ratio search over {$\frac{1}{4}$, $\frac{1}{3}$, $\frac{1}{2}$, $\frac{3}{4}$} and apply the approach to the DrugCombDB dataset. The obtained results are given in Table 5. As it is shown, when the overlap stride ratio is set to ½, the proposed approach in all patch size values gets better performance. When the overlap stride ratio is low, it means that two neighboring patches have a low overlap with each other. Moreover, the best AUPR value is obtained at a patch size of 40.

**Table 5.** Investigate the effect of the patch size and overlap stride ratio on the DrugCombDB dataset^a^.

Overlap ratio	$\frac{1}{4}$	$\frac{1}{3}$	$\frac{1}{2}$	$\frac{3}{4}$
Patch size				
20	0.8159	0.8148	0.8314	0.8294
30	0.8203	0.8214	0.8458	0.8342
40	0.8211	0.8209	0.8487	0.8417
50	0.8158	0.8180	0.8358	0.8321
60	0.8121	0.8125	0.8268	0.8217

a AUPR is reported as the performance measure.

In the following, we have designed another experiment to investigate how the proposed approach could predict synergy scores for new unseen data. To do so, for the DrugCombDB dataset, we have utilized the leave-one-cell-line-out setting. In this case, for a cell line, whole related samples are excluded and then training is done and then the trained model is applied to the excluded samples. This procedure is done for whole cell lines and results are averaged. The obtained results are shown in Table 6. As is shown in all measures except AUC-ROC, the proposed approach performs better than the other approaches.

**Table 6.** The comparison of the DeepTraSynergy with the comparable on the DrugCombDB dataset in the leave-one-cell-line-out setting.

Model	ACC	Recall	AUC-ROC	AUC-PR	F1	*P*-value *t*-test
KGNN	NA	NA	NA	NA	NA	—
GCN^a^	0.541	0.692	0.584	0.461	0.506	6.39 × 10^−2^
GraRep	NA	NA	NA	NA	NA	—
DeepSynergy	0.583	0.743	0.630	0.450	0.513	1.22 × 10^−1^
DeepDDS	0.790	0.531	**0.812**	0.676	0.593	3.57 × 10^−2^
GraphSynergy^a^	0.798	0.591	0.810	0.698	0.632	5.92 × 10^−3^
DFFNDDS	0.799	0.572	0.829	0.693	0.619	8.27 × 10^−2^
NEXGB	NA	NA	NA	NA	NA	—
**DeepTraSynergy**	**0.812**	**0.610**	0.817	**0.715**	**0.658**	

a We run the approach by ourselves.

The bolded items in the table are the performance measure of our model.

#### 3.4.2 Evaluation of independent test set

**Figure 4.**
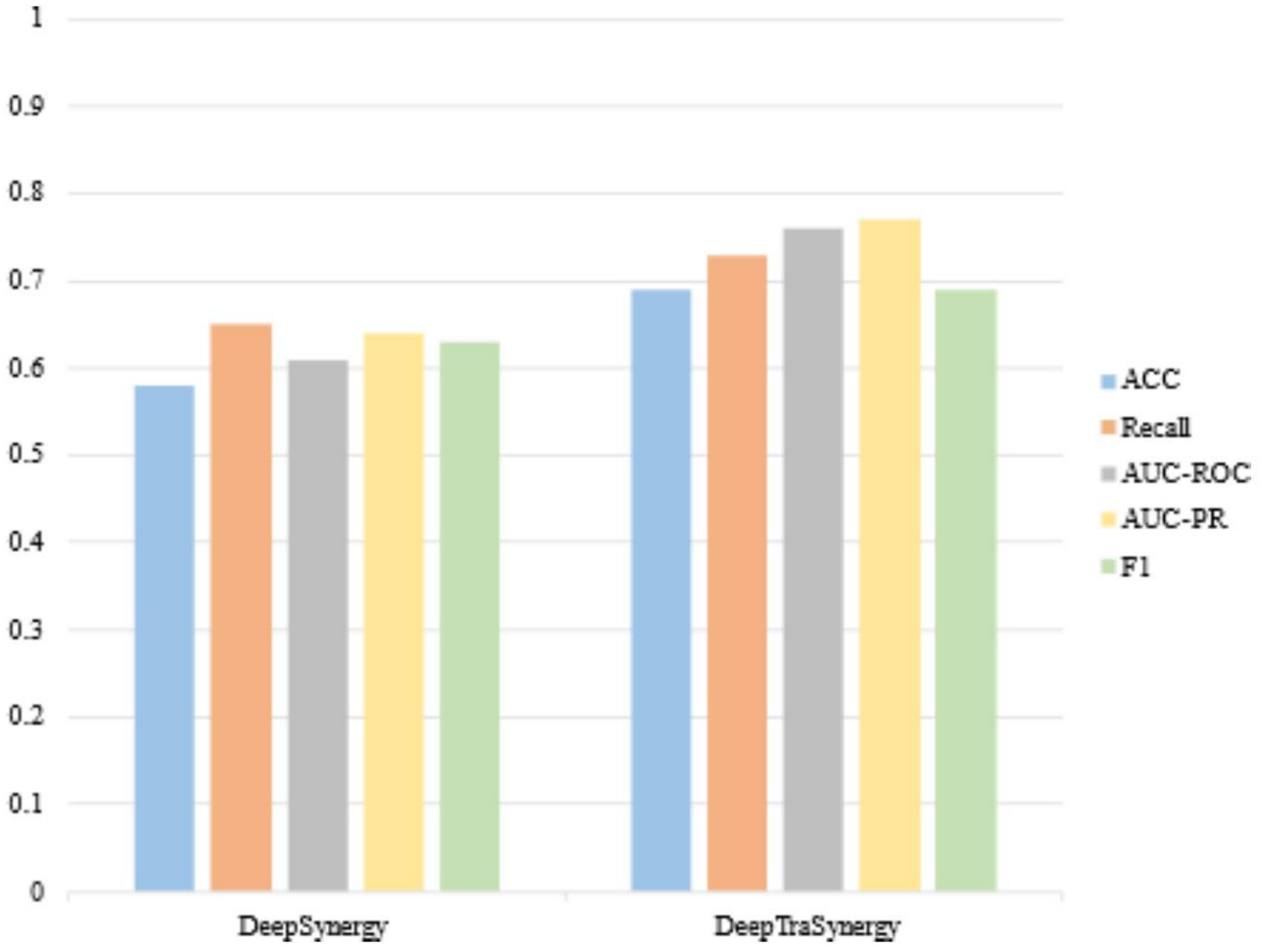
The comparison of DeepTraSynergy with DeepSynergy in the evaluation of an independent dataset.

In this section, the goal is to evaluate the generalization ability of the proposed approach. To do so, we have designed another experiment. In this experiment, we have trained the model on a dataset and applied it to an independent dataset. In this case, the model is trained on the O’Neil dataset and is evaluated on the DrugCombDB dataset. The reason is that DrugCombDB is bigger than the O’Neil dataset and there are some cell lines and drugs that do not exist in the O’Neil dataset. Table 7 shows the obtained results. The obtained results are compared with the model when it is trained and tested on the same dataset (i.e. the DrugCombDB dataset). By comparing the obtained results in Table 7 with the reported results of the state-of-the-art methods in Table 3, we have found out that the proposed method achieves comparable results with many of them like DeepSynergy, GraRep, and KGNN. It verifies the generalizability of the proposed approach. To compare the generalization ability with the state-of-the-art methods, we have downloaded the provided source code of GraphSynergy and DeepSynergy and then we run the code with the same setting. After running, GraphSynergy’s result for all measures was zero and it is because of its algorithm. The comparison between DeepSyergy and DeepTraSynergy is given in Fig. 4.

**Table 7.** The obtained result of DeepTraSynergy in an independent test set^a^.

Model	ACC	Recall	AUC-ROC	AUC-PR	F1
DeepTraSynergy (trained on O’Neil)	0.6941	0.7309	0.7583	0.7659	0.6926
DeepTraSynergy (trained on DrugCombDB)	0.7715	0.8012	0.8321	0.8487	0.7608

a DeepTraSynergy is evaluated on the DrugCombDB dataset.

#### 3.4.3 Performance evaluation by hyperparameter tuning

To show how the proposed approach is sensitive to hyperparameter tuning, we have done another experiment. In this case, we have two scenarios: (i) we tune the hyperparameter values on the DrugCombDB dataset and we train and test the model on the DrugCombDB dataset. (ii) We tune the hyperparameter values on the OncologyScreen dataset and we train and test the model on the DrugCombDB dataset. The obtained results are shown in Table 8—DrugCombDB. As is shown, there is a small difference between the results but it is not too significant. Also, we have done another experiment. In this case, first, we tune the hyperparameter values on the OncologyScreen dataset and we train and test the model on the OncologyScreen dataset. Then, we tune the hyperparameter values on the DrugCombDB dataset and we train and test the model on the OncologyScreen dataset. The obtained results are shown in Table 8—OncologyScreen. In this case, too, the outcome shows a slight discrepancy between the results.

**Table 8.** The obtained result of DeepTraSynergy evaluation by hyperparameter tuning.

DrugCombDB					
Model	ACC	Recall	AUC-ROC	AUC-PR	F1
DeepTraSynergy (hyperparameter tuning on DrugCombDB dataset)	0.7613	0.7981	0.8411	0.8459	0.7532
DeepTraSynergy (hyperparameter tuning on OncologyScreen dataset)	0.7715	0.8012	0.8321	0.8487	0.7608
OncologyScreen					
DeepTraSynergy (hyperparameter tuning on DrugCombDB dataset)	0.8013	0.8147	0.8502	0.8624	0.8029
DeepTraSynergy (hyperparameter tuning on OncologyScreen dataset)	0.8052	0.8491	0.8637	0.8818	0.8112

#### 3.4.4 Predicting novel synergistic combinations

In this section, we have designed an experiment to predict a novel synergistic combination using DeepTraSynergy. In this case, the O’Neil drug combination dataset is used to train the model. To generate candidate drug combinations, like DeepDDS (Wang *et al.* 2021), 42 FDA-approved drugs are selected to generate 855 candidate drug pairs. The top ten predicted drug combinations on the A375 cancer cell line (human melanoma cell lines) are given in Table 9. We have done a literature search and found the literature confirmation for at least five of them. We have given the reference to these related publications in Table 9. As it is shown, Temsirolimus exists in seven of ten predicted combinations. The reason is that it is an antineoplastic agent used in the treatment of renal cell carcinoma. By comparing the reported result of DeepDDS and DeepTraSynergy, we found out that only one combination, Copanlisib, and Regorafenib, is common among the top ten predicted synergistic combinations of the two approaches. For some combinations, we did not find any literature confirmation but for example, in Axitinib and Temsirolimus combinations, Axitinib has demonstrable single-agent activity in melanoma (Fruehauf *et al.* 2008). Also, it is shown that Talazoparib in combination with Niraparib could treat melanoma cells (Jonuscheit *et al.* 2021). It should be noted that we have provided the top 100 highly scored predictions as an Excel file in Supplementary Data.

**Table 9.** Top 10 predicted synergistic combinations on A375 cancer cell line.

Drug A	Drug B	Cell line	Publications
Everolimus	Temsirolimus	A375	(Di Lorenzo *et al.* 2011, Iacovelli *et al.* 2015, Liu *et al.* 2022)
Temsirolimus	Cabozantinib	A375	NA
Copanlisib	Regorafenib	A375	(Wang *et al.* 2022)
Pazopanib	Temsirolimus	A375	(Semrad *et al.* 2013, Kwon *et al.* 2017)
Everolimus	Pazopanib	A375	(Wagle *et al.* 2014, Grivas *et al.* 2019)
Venetoclax	Pazopanib	A375	NA
Regorafenib	Temsirolimus	A375	NA
Talazoparib	Temsirolimus	A375	NA
Axitinib	Temsirolimus	A375	NA
Sorafenib	Temsirolimus	A375	(Davies *et al.* 2012, Margolin *et al.* 2012, Rangwala *et al.* 2014)

## 4 Discussion

This paper presents a new method for predicting drug synergies. The contributions of the proposed approach are utilizing transformers as feature extractors and proposing a new architecture that uses auxiliary knowledge like protein–protein interaction network, compound–protein interaction, and cell–protein interaction. Transformer-based feature extractor simultaneously captures the local structure and encodes the long-range dependencies. Since a limited number of drug combinations and drug–protein interactions for each specific cell line exist, some hidden connections in the network may be obscure. To meet this problem, an architecture is designed in the proposed approach that incorporates two other auxiliary knowledge, including drug–protein interaction and toxicity prediction, for drug synergy prediction. The ablation study results and comparing results with state-of-the-art approaches confirm that the transformer-based feature extractor (without utilizing any other knowledge) learns more discriminative features for drug molecules. Moreover, it can be seen from the experiment that incorporating a compound–protein interaction network in the proposed approach can improve the results.

To evaluate the generalization ability of the proposed approach, we have evaluated the learned model on an independent dataset and the obtained results confirm that the proposed approach has a good generalizability. Also, by predicting new synergistic combinations for the A375 cell line, we have found out that in the top ten predicted pairs, at least five of them have literature confirmation.

For future work, we seek to find a better way to learn the compound–protein interaction network. One of the main contributions of the proposed approach is that it shows that the drug–protein interaction network plays an important role in drug synergy prediction. Moreover, not only drug–protein interaction but also how drugs bind to the protein or proteins (in the same cell line) provide too meaningful information for drug synergy prediction. Hence, providing pockets that drug could bind them and incorporating this information in drug synergy prediction could improve drug synergy prediction performance. Moreover, the way of combining knowledge of different modalities could be enhanced using attention-based fusion techniques.

## Supplementary Material

btad438_Supplementary_Data
